# Immunogenetics associated with severe coccidioidomycosis

**DOI:** 10.1172/jci.insight.159491

**Published:** 2022-11-22

**Authors:** Amy P. Hsu, Agnieszka Korzeniowska, Cynthia C. Aguilar, Jingwen Gu, Eric Karlins, Andrew J. Oler, Gang Chen, Glennys V. Reynoso, Joie Davis, Alexandria Chaput, Tao Peng, Ling Sun, Justin B. Lack, Derek J. Bays, Ethan R. Stewart, Sarah E. Waldman, Daniel A. Powell, Fariba M. Donovan, Jigar V. Desai, Nima Pouladi, Debra A. Long Priel, Daisuke Yamanaka, Sergio D. Rosenzweig, Julie E. Niemela, Jennifer Stoddard, Alexandra F. Freeman, Christa S. Zerbe, Douglas B. Kuhns, Yves A. Lussier, Kenneth N. Olivier, Richard C. Boucher, Heather D. Hickman, Jeffrey Frelinger, Joshua Fierer, Lisa F. Shubitz, Thomas L. Leto, George R. Thompson, John N. Galgiani, Michail S. Lionakis, Steven M. Holland

**Affiliations:** 1 Laboratory of Clinical Immunology and Microbiology, National Institute of Allergy and Infectious Diseases (NIAID), NIH, Bethesda, Maryland, USA.; 2 Department of Cell Biology and Molecular Genetics, University of Maryland, College Park, Maryland, USA.; 3 Bioinformatics and Computational Biosciences Branch, Office of Cyber Infrastructure and Computational Biology, NIAID, NIH, Bethesda, Maryland, USA.; 4 Marsico Lung Institute and Cystic Fibrosis Research Center, University of North Carolina at Chapel Hill, Chapel Hill, North Carolina, USA.; 5 Valley Fever Center for Excellence, University of Arizona College of Medicine–Tucson, Tucson, Arizona, USA.; 6 Department of Respiratory and Critical Care Medicine, Laboratory of Pulmonary Immunology and Inflammation, West China Hospital, Sichuan University, Chengdu, Sichuan Province, China.; 7 NIAID Collaborative Bioinformatics Resource, NIAID, NIH, Bethesda, Maryland, USA.; 8 Advanced Biomedical Computational Science, Frederick National Laboratory for Cancer Research, Leidos Biomedical Research, Inc., Frederick, Maryland, USA.; 9 Department of Internal Medicine, Division of Infectious Diseases, UC Davis Health, Sacramento, California, USA.; 10 Department of Immunobiology, University of Arizona, Tucson, Arizona, USA.; 11 Department of Medicine, University of Arizona College of Medicine–Tucson, Tucson, Arizona, USA.; 12 Center for Biomedical Informatics and Biostatistics and; 13 The Center for Applied Genetics and Genomic Medicine, Department of Medicine, University of Arizona, Tucson, Arizona, USA.; 14 Neutrophil Monitoring Laboratory, Applied/Developmental Research Directorate, Leidos Biomedical Research, Inc, Frederick National Laboratory for Cancer Research, Frederick, Maryland, USA.; 15 Laboratory for Immunopharmacology of Microbial Products, School of Pharmacy, Tokyo University of Pharmacy and Life Sciences, Hachioji, Tokyo, Japan.; 16 Immunology Service, Department of Laboratory Medicine, Clinical Center and; 17 Laboratory of Chronic Airway Infection, Pulmonary Branch, National Heart, Lung, and Blood Institute, NIH, Bethesda, Maryland, USA.; 18 Department of Immunobiology, University of Arizona, Tucson, Arizona, USA.; 19 VA HealthCare San Diego, San Diego, California, USA.; 20 Division of Infectious Diseases, Departments of Pathology and Medicine, School of Medicine, University of California San Diego, La Jolla, California, USA.; 21 Department of Medical Microbiology and Immunology, University of California Davis, Davis, California, USA.

**Keywords:** Genetics, Infectious disease, Fungal infections, Innate immunity, Population genetics

## Abstract

Disseminated coccidioidomycosis (DCM) is caused by *Coccidioides*, pathogenic fungi endemic to the southwestern United States and Mexico. Illness occurs in approximately 30% of those infected, less than 1% of whom develop disseminated disease. To address why some individuals allow dissemination, we enrolled patients with DCM and performed whole-exome sequencing. In an exploratory set of 67 patients with DCM, 2 had haploinsufficient *STAT3* mutations, and defects in β-glucan sensing and response were seen in 34 of 67 cases. Damaging *CLEC7A* and *PLCG2* variants were associated with impaired production of β-glucan–stimulated TNF-α from PBMCs compared with healthy controls. Using ancestry-matched controls, damaging *CLEC7A* and *PLCG2* variants were overrepresented in DCM, including *CLEC7A* Y238* and *PLCG2* R268W. A validation cohort of 111 patients with DCM confirmed the *PLCG2* R268W, *CLEC7A* I223S, and *CLEC7A* Y238* variants. Stimulation with a DECTIN-1 agonist induced DUOX1/DUOXA1–derived hydrogen peroxide [H_2_O_2_] in transfected cells. Heterozygous *DUOX1* or *DUOXA1* variants that impaired H_2_O_2_ production were overrepresented in discovery and validation cohorts. Patients with DCM have impaired β-glucan sensing or response affecting TNF-α and H_2_O_2_ production. Impaired *Coccidioides* recognition and decreased cellular response are associated with disseminated coccidioidomycosis.

## Graphical abstract

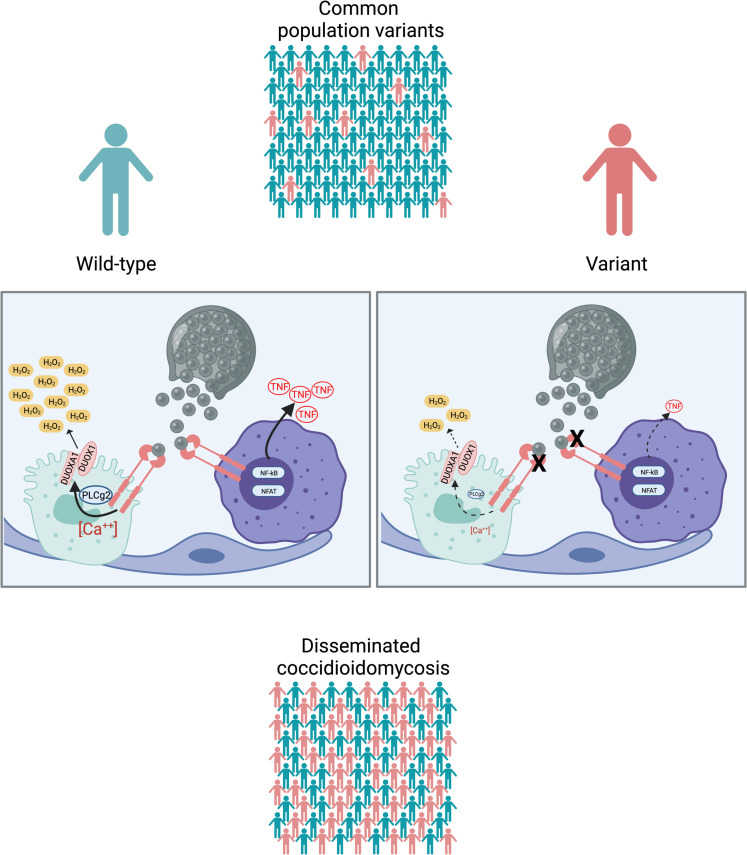

## Introduction

Coccidioidomycosis, or valley fever, is caused by infection with the soil-dwelling, dimorphic fungi, *Coccidioides immitis* and *C*. *posadasii*, prevalent in the southwestern United States, Mexico, and Central and South America. Infection occurs after inhalation of arthroconidia, which morph into spherules that release hundreds of endospores 3 to 5 days later, becoming new spherules and establishing infection. Approximately one-third of the 150,000 US individuals infected annually will develop a symptomatic, self-limited pneumonia, while less than 1% of those will develop disseminated disease (1–3) mostly affecting meninges, bones, skin, or joints (4). The median hospitalization costs are more than US$70,000 (5); 2017 costs in California surpassed $700 million (6) and total lifetime costs for 2019 Arizona cases were estimated to be $736 million (7).

Similar to *Mycobacterium tuberculosis*, *Coccidioides* can cause illness in otherwise healthy hosts. Infection is more likely to disseminate in immunocompromised patients, including people with AIDS; those receiving chemotherapy, organ transplants, or immunomodulatory biologics (e.g., anti-TNF and anti–IL-6); or women in the third trimester of pregnancy (8). Interestingly, only a few patients with DCM have identified mutations, and these are all within the IL-12/IFN-γ and signal transducer and activator of transcription-3 (STAT3) pathways (8). Therefore, we used whole-exome sequencing to explore genetic risk factors in disseminated coccidioidomycosis.

## Results

### Description of cohort.

We enrolled 67 patients with DCM, predominantly from Arizona, in an exploratory cohort (Tables 1–3). Patients with histories of other invasive fungal infections, HIV infection, or receiving immunosuppression were excluded. Median lymphocyte count was 1.8 × 10^3^/μL (range, 1.28 × 10^3^/μL to 4.3 × 10^3^/μL), median monocyte count was 0.53 × 10^3^/μL (range, 0.3 × 10^3^/μL to 0.74 × 10^3^/μL). Consistent with previous reports (8, 9), most patients were male (*n* = 44; 65.7%). Median age at enrollment was 41 years (range, 10 months to 85 years). The cohort included individuals with the following genetically determined ancestries: European (*n* = 20), admixed American and Latino (*n* = 20), African or African American (*n* = 18), East Asian (*n* = 4), South Asian (*n* = 3), and 2 people whose ancestry was indeterminate. Because *Coccidioides* exhibits tissue tropisms, we classified dissemination sites as bone (*n* = 13), CNS (*n* = 28), or soft tissue (*n* = 17). There were 9 patients with dissemination to 2 different tissues (*n* = 6 bone and soft tissue; *n* = 1 bone and CNS; *n* = 2 CNS and soft tissue). Forty patients reported other previous infections, including 15 with noninvasive fungal infections (*n* = 11 dermatophytoses), 27 with viral infections (*n* = 7 with shingles), and 3 with bacterial infections, (nontuberculous mycobacteria, *Staphylococcus*, and *Salmonella*) (Tables 1 and 2). Twelve patients reported recurrent upper respiratory infections, and 7 reported asthma. There were 27 patients without previous invasive infections (Table 3).

### Monogenic mutations.

To date, only 12 patients with DCM have been described who carried mutations associated with primary immunodeficiencies, all within the IL-12/IFN-γ and STAT3 axes (8, 10, 11). Initial analysis of our patients considered rare, damaging variants, with a focus on the International Union of Immunological Societies list of genes associated with human inborn errors of immunity (12) by which 2 patients were identified with novel nonsense mutations in *STAT3* (c.250C>T p.R84* and c.1267C>T p.R423*). No biallelic or dominant mutations were identified in *IL12RB1*, *IFNGR1*, *STAT1, STAT4,* or *GATA2*. Although the first identified monogenic mutation leading to DCM was identified in a patient with dominant-negative STAT3 leading to autosomal-dominant hyper-IgE syndrome (AD-HIES; ref. 13), the 2 STAT3-haploinsufficient patients did not exhibit AD-HIES characteristics. Similar to the single, previously reported patient with STAT3 haploinsufficiency (14), these 2 patients had unremarkable clinical histories until DCM; both patients developed fatal CNS dissemination.

### Identification of DECTIN-1 pathway mutations.

**Figure 1:**
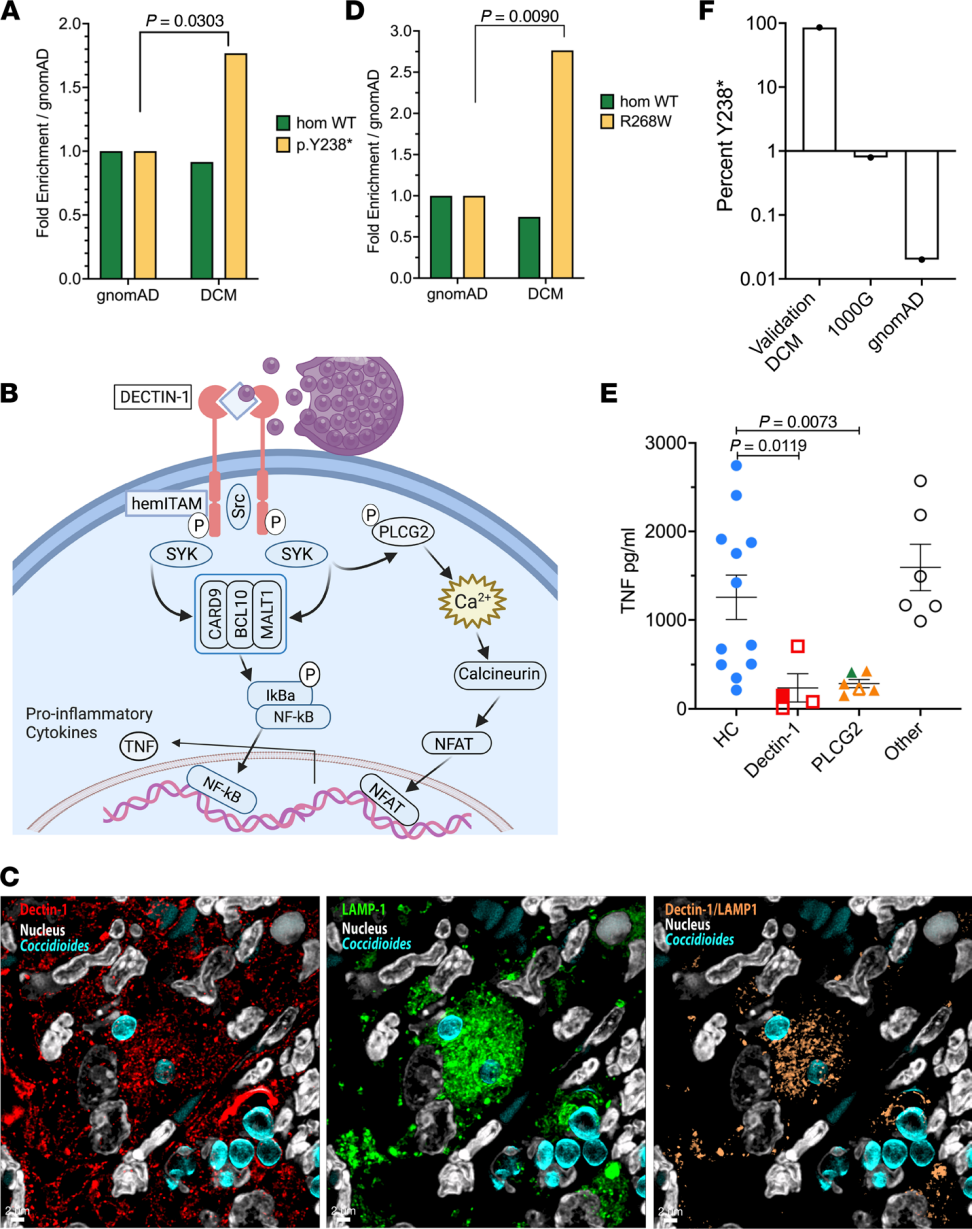
Fungal recognition variants in patients with DCM. (**A**) Fold-enrichment of *CLEC7A*, c.714T>G; p.Y238* variant in patients with DCM compared with the gnomAD. Normalized to frequency of homozygous WT or variant carriers in gnomAD. *P* = 0.0303, Fisher’s exact test. (**B**) Parallel signaling pathways after β-glucan recognition by DECTIN-1 leading to activation of NF-κB and NFAT transcription factors and production of TNF-α. The figure was created using BioRender. (**C**) Confocal microscopy of lung from *C*. *posadasii*–infected C57BL/6 mouse showing DECTIN-1 (red) localized near endospores (blue) (left), LAMP-1 (green) localization near endospores (middle), and colocalization of DECTIN-1 and LAMP-1 (tan) around endospores (right). (**D**) Frequency of patients of European ancestry with DCM with *PLCG2*, c.802C>T; p.R268W genotype normalized to the non–Finnish European population in gnomAD. *P* = 0.0077 Fisher’s exact test. (**E**) Particulate β-glucan–induced TNF production by PBMCs from patients (*n* = 16) or healthy control (HC) participants (*n* = 12). DECTIN-1 variants (*n* = 4) include homozygous p.Y238* (filled) and heterozygous p.Y238* (open). Patients withPLCG2 variants (*n* = 6) include p.R268W heterozygotes (yellow symbols), p.M28L heterozygotes (green symbol), and p.R268W and p.K775R compound heterozygotes (open yellow symbol). Patients in the “other” category (*n* = 6) lack identified causal variants. *P* values were calculated using Brown-Forsythe and Welch ANOVA with Dunnett’s T3 multiple comparisons test. (**F**) Frequency of Y238* among East Asian patients from DCM validation cohort compared with the 1000G and gnomAD.

Given the paucity of identified monogenic mutations and the limited geographic presence of *Coccidioides*, we examined our DCM cohort for more common variants. We filtered our whole-exome sequencing data using a 10% minor-allele frequency cutoff and combined annotation-dependent depletion (CADD) score greater than 20 as a prediction of deleteriousness. Hypothesizing that even common defects in fungal recognition could be important, we specifically queried *CLEC7A* c.714T>G; p.Y238*. *CLEC7A* encodes DECTIN-1, the C-type lectin pattern recognition receptor for the fungal cell-wall component β-glucan. DECTIN-1 mediates innate fungal recognition, and p.Y238* is associated with familial mucocutaneous candidiasis (15), increased susceptibility to invasive aspergillosis after hematopoietic stem cell transplantation (HSCT) (16), and chronic lung allograft dysfunction after lung transplantation (17). We found homozygous *CLEC7A* c.714T>G; p.Y238* in 3 of 67 patients with DCM (4.5%) compared with 680 of 141,265 (0.48%) in the reference Genome Aggregation Database [gnomAD, version 2.1 (18)] (*P* = 0.0027, Fisher’s exact test) (Supplemental Figure 1; supplemental material available online with this article; https://doi.org/10.1172/jci.insight.159491DS1). Additionally, 10 patients with DCM were heterozygous for p.Y238*. Therefore, 13 of 67 patients with DCM (19.4%) carried p.Y238*, a significantly percentage than that found in gnomAD (*n* = 16,450 of 141,265 [11.6%]; *P* = 0.0303, Database [gnomAD, version 2.1 (18)] (*P* = 0.0027, Fisher’s exact test) (Supplemental Figure 1; supplemental material available online with this article; https://doi.org/10.1172/jci.insight.159491DS1). Additionally, 10 patients with DCM were heterozygous for p.Y238*. Therefore, 13 of 67 patients with DCM (19.4%) carried p.Y238*, a significantly percentage than that found in gnomAD (*n* = 16,450 of 141,265 [11.6%]; *P* = 0.0303, Fisher’s exact test) (Figure 1A). p.Y238* results in loss of the terminal 10 amino acids of the C-type lectin domain of DECTIN-1, including the key structural residue, Cys241, which forms a disulfide bridge with Cys147 (19) (Supplemental Figure 2).

Fungal recognition by DECTIN-1 is the first step in a signaling cascade (Figure 1B) that activates SRC kinase, leading to phosphorylation of spleen tyrosine kinase (SYK), assembly of the CARD9-BCL10-MALT1 complex, and NF-κB activation. Concurrently, SYK phosphorylates PLCγ2, increasing intracellular Ca^++^ concentration, and inducing non–CARD9-dependent NFAT pathways (20–22). Upon engagement, DECTIN-1 is endocytosed (23). Although the 70 to 100 micron size of spherules prohibits engulfment, endospores recognized by DECTIN-1 are phagocytosed, localizing to DECTIN-1/LAMP-1–positive phagolysosomes (Figure 1C). In view of the *CLEC7A* Y238* mutation abundance in our DCM cohort, we screened for additional damaging variants in this fungal recognition pathway. One patient had heterozygous DECTIN-1 p.I223S, and 15 patients carried predicted damaging *PLCG2* (encoding PLCγ2) variants. *PLCG2* p.R268W was seen in 9 of the 67 patients, including 3 with *CLEC7A* p.Y238*. Seven of the 9 individuals with *PLCG2* p.R268W were among the 20 Europeans in our cohort, significantly more than seen in the non–Finnish European gnomAD population (*n* = 7 of 20 [35%] vs. 7917 of 62,273 [12.7%]; *P* = 0.009, Fisher’s exact test) (Figure 1D).

### Decreased TNF-α production in response to β-glucan.

To identify relevant biologic effects of these variants, PBMCs from patients and healthy control participants were stimulated with purified, particulate β-glucan, a DECTIN-1 agonist, or LPS, a bacterial component that signals through TLR4. In response to LPS, all patients produced normal levels of TNF-α (Supplemental Figure 3A). However, in response to β-glucan, cells from patients with either heterozygous or biallelic DECTIN-1 pathway variants produced significantly less TNF-α than those of control participants (Figure 1E). DECTIN-1 p.Y238* homozygous (filled symbol in Figure 1E) and heterozygous (open symbols in Figure 1E) patient cells showed similar defects in TNF-α production, suggesting that DECTIN-1 p.Y238* is dominant negative (19, 24). Patient cells tested, including *PLCG2* R268W and M28L, were impaired in β-glucan–induced TNF-α production, whereas production of IFN-γ, IL-12p70, and IL-17 did not differ between patients and healthy control participants (Supplemental Figures 3, B–D). Two control samples from the NIH blood bank in Maryland also produced minimal TNF-α, suggesting these individuals might also be at risk if exposed. These data show that cells from patients carrying DECTIN-1 pathway variants produced significantly less TNF-α than those of control participants (*P* < 0.005; Supplemental Figure 2E) in response to β-glucan while retaining normal IFN-γ, IL-12p70, and IL-17 production and normal responses to LPS.

### Variant burdens in validation and reference cohorts.

Given the mixed population of our cohort, we used ancestry-matched controls from the 1000 Genomes Project (1000G) and a generalized linear model including ancestry principal components. Variant burden across genes and for recurrent variants within the DECTIN-1 signaling pathway were evaluated. Patients with DCM carried more damaging DECTIN-1 variants (*P* = 0.0206; OR, 3.45 [95% CI, 1.21–9.84]). Specifically, the Y238* variant was seen in 13 members of our cohort (*P* = 0.0105; OR, 4.05 [95% CI, 1.39–11.84]). Damaging variants across *PLCG2* were also overrepresented (*P* = 0.015; OR, 3.05 [95% CI, 1.24–7.51]), including R268W (*P* = 0.0025; OR, 5.46 [95% CI, 1.82–16.37]) and N571S (*P* = 0.0166; OR 26.22 [95% CI, 1.81–379.37]). Within our cohort of 67 patients, 24 (35.8%) carried at least 1 heterozygous variant in the DECTIN-1/PLCγ2 signaling pathway.

To validate our exploratory analysis, we analyzed an independent DCM cohort (Supplemental Tables 1–3) consisting of patients with meningitis (*n* = 31; mean age, 54 [range, 24–75] years) or without meningitis (*n* = 80; mean age, 51 [range, 21–86] years), predominantly from California. Patient ancestry was determined using principal component analysis (PCA) and the 1000G as a reference. Previously identified monogenic susceptibility genes *IL12RB1, IFNGR1, IFNGR2, STAT1, STAT4,* and *STAT3* were screened, and no causative mutations were found.

In the DCM validation cohort, specific variants in both *CLEC7A* and *PLCG2* were again overrepresented: *CLEC7A* I223S (*P* = 0.0444; OR, 3.44 [95% CI, 1.03–11.50]) and *PLCG2* R268W (*P* = 0.0276; OR, 2.49 [95% CI, 1.11–5.59]). In the overall validation cohort, CLEC7A Y238* did not reach significance; however, 100% of East Asian patients with DCM carried DECTIN-1 pathway variants. These DECTIN-1 pathway variants included Y238* (*n* = 12 of 14; 85.7%), I223S (*n* = 1 of 14), and PLCG2 R268W (*n* = 1 of 14). In contrast, only 4 of 504 East Asians (0.79%) in 1000G (*P* < 0.0001) and 2 of 9973 (0.02%) of East Asians in gnomAD (*P* < 0.0001) carried Y238* (Figure 1F). Among the East Asian individuals with DECTIN-1 Y238*, 1 also carried PLCG2 M28L.

Increased risk of dissemination among African Americans and East Asians has been documented extensively (25). Multivariate analysis of coccidioidomycosis in Kern County, California, comparing patients with mild coccidioidomycosis with those with DCM showed an increased risk among African Americans to develop disseminated disease (OR, 4.6 [95% CI, 1.4–15]) (26). Hospitalization data from Arizona and California during 2005–2011 documented increased incidence of hospitalization for coccidioidomycosis among African Americans (8.1 [in 2005] to 16.1 [in 2011] per 100,000) and Asian/Pacific Islanders (8.71 [in 2005] to 14.72 [in 2011] per 100,000) compared with other ethnicities (3.53 [in 2005] to 7.92 [in 2011] per 100,000) (27). Within our validation cohort, presentation of disease by ancestry is consistent with the epidemiologic data, with 80% of African Americans in the cohort presenting with DCM. In contrast, 75% to 80% of those with European or admixed American ancestry presented with primary pulmonary disease (Supplemental Figure 4). These disparities suggest underlying factors affecting fungal control, such as the ones we have demonstrated among the East-Asians within our cohort.

### Nonhematopoietic fungal recognition.

Heterozygous DECTIN-1 p.Y238* in either donors or recipients is associated with invasive aspergillosis after HSCT (16), demonstrating both hematopoietic and nonhematopoietic contributions of DECTIN-1 p.Y238* to antifungal defense. DECTIN-1 knockdown also impaired β-glucan–induced cytokine production in a pulmonary epithelial cell line (16). We next considered some downstream effects of DECTIN-1 activation. Several NADPH oxidases in the NOX/DUOX family participate in innate immunity by generation of ROS (superoxide or hydrogen peroxide [H_2_O_2_]) after stimulation. Recognizing that DECTIN-1 engagement on neutrophils produces superoxide via NADPH–oxidase complex (28), we examined the pulmonary epithelial NADPH–oxidase complex DUOX1/DUOXA1.

**Figure 2:**
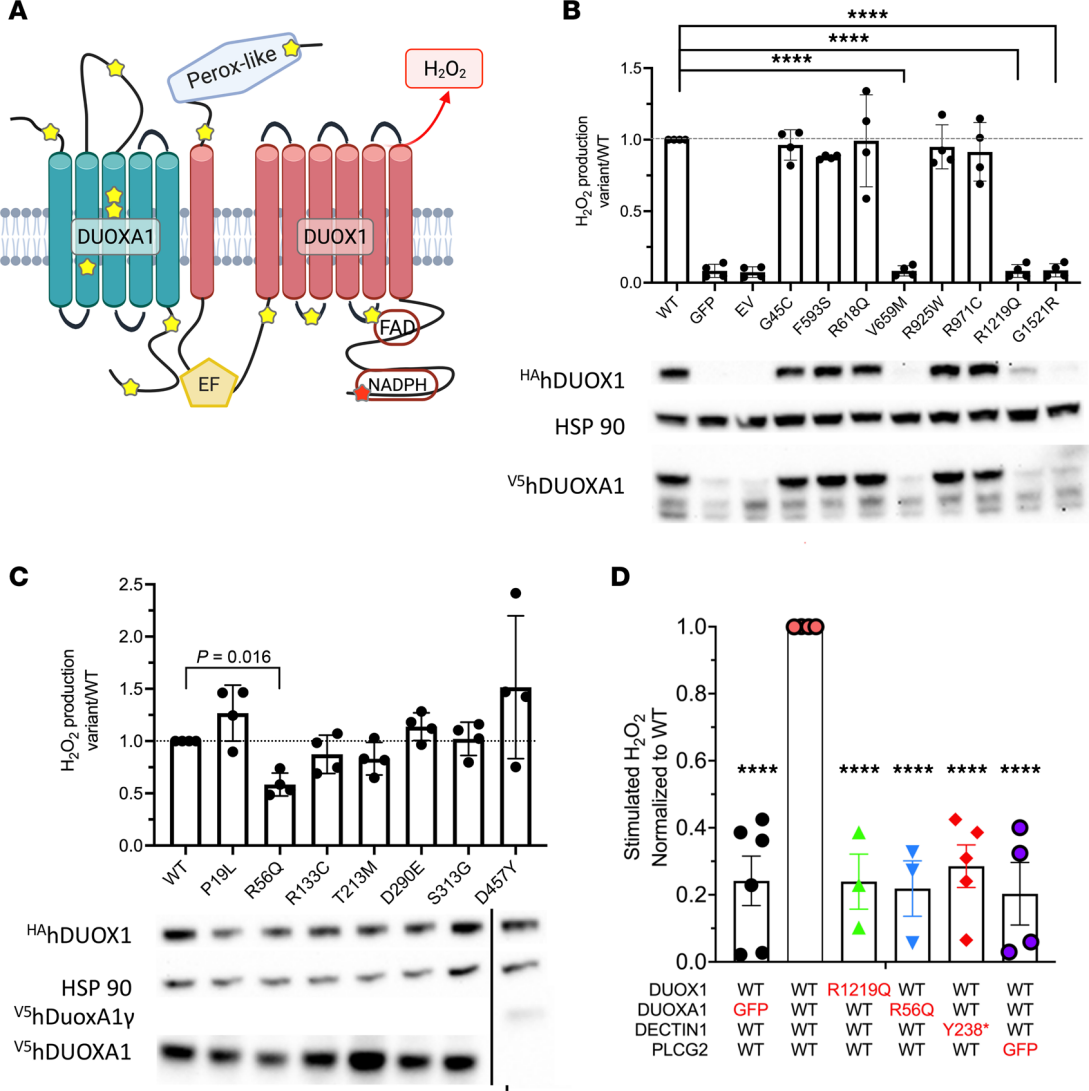
DECTIN-1 signaling drives H_2_O_2_ production by DUOX1 and DUOXA1 in HEK cells. (**A**) DECTIN-1–activated PLCγ2 releases intracellular Ca^++^, which activates the EF-hand domains of DUOX1, leading to H_2_O_2_ production. DUOX1 and DUOXA1 patient variants are highlighted by yellow stars. (**B**) DUOX1 variants identified in patients with DCM were transfected into HEK Flp-In cells stably expressing WT DUOXA1. Cells were stimulated with ionomycin, and H_2_O_2_ production was measured for 60 minutes. Results are the average of triplicate wells presented as the ratio of H_2_O_2_ production by variant or WT; each dot represents the average of triplicate wells from a unique experiment. ****P < 0.0001 by ordinary 1-way ANOVA and Dunnett’s multiple comparisons test. Western blot showing decreased protein abundance of several DUOX1 variants after transfection. (**C**) DUOXA1 variants, identified in patients with DCM, transfected into HEK Flp-In cells stably expressing DUOX1. Measured as in **B**. *P* = 0.016 by ordinary 1-way ANOVA and Dunnett’s multiple comparisons test. (**D**) Hydrogen peroxide production in HEK cells transfected with WT or patient variant DUOX1, DUOXA1, or DECTIN1, or lacking PLCG2 constructs. Results are the average of triplicate wells presented as the ratio of H_2_O_2_ production by variant/WT; each dot represents a unique experiment. ****P < 0.0001 using ordinary 1-way ANOVA and Dunnett’s multiple comparisons test.

Heterodimeric assembly of DUOX1 and its obligate accessory maturation factor, DUOXA1, in the ER enables its transit to the apical surface of specific epithelial cells (29). DUOX1 releases H_2_O_2_ in response to calcium-mobilizing agonists because of its 2 intracellular Ca^++^-sensing EF-hand domains that activate its NADPH oxidase activity. *Duox1*^–/–^ mice have increased morbidity and mortality when influenza challenged (30). Whereas bi-allelic mutations in *DUOX2* and *DUOXA2* cause congenital hypothyroidism, heterozygous *DUOX2* variants have been implicated in inflammatory bowel disease (IBD) (31). Similar to DUOX2/DUOXA2 in IBD, variants were found throughout DUOX1 and DUOXA1 in our exploratory cohort (Figure 2A, yellow stars).

In contrast to the recurrent DECTIN-1 and PLCG2 variants, DUOX1/DUOXA1 variants were mostly identified in single patients and were found in 10 of 18 African Americans (55.6%), 3 of 20 non-Hispanic Europeans (15.0%), 1 of 20 admixed Americans/Latinos (5.0%), and 1 of 3 South Asians (33.3%). In the validation cohort, 10 of 48 African Americans (20.8%) carried damaging *DUOX1/DUOXA1* variants compared with 1 of 14 Europeans (7.1%), 4 of 34 admixed Americans/Latinos (11.8%), and 2 of 14 East Asians (14.3%). For each individual variant we compared the cohort frequency to the gnomAD, version 2.1, population frequency corresponding to patient ancestry (Supplemental Table 4). In the discovery cohort, 10 of 13 variants had a gnomAD population-specific frequency of less than 2.5 per 1000 with 7 of 13 being less than 1 per 1000. The frequency of these rare alleles was higher in both the discovery and validation cohorts compared with either gnomAD, version 2.1, ancestry-specific population frequency or the gnomAD, version 2.1, frequency for all populations (Supplemental Figure 5).

To evaluate the identified DUOX1/DUOXA1 variants, Flp-In 293 cells stably expressing WT DUOXA1 were transfected with HA-tagged DUOX1; 3 DUOX1 variants exhibited decreased protein stability and did not produce H_2_O_2_ after stimulation with ionomycin, despite overexpression (Figure 2B). Similarly, Flp-In 293 cells expressing DUOX1 were transfected with V5-tagged DUOXA1 variants. One identified variant, p.R56Q, previously reported to have compromised H_2_O_2_ production (32), showed significantly decreased H_2_O_2_ production (Figure 2C).

Given the expression of DECTIN-1 by bronchial epithelial cells (33), we hypothesized that DECTIN-1 activation by fungal components and subsequent PLCγ2-dependent intracellular Ca^++^ increase might activate DUOX1 through its Ca^++^-sensing EF-hand domains, causing H_2_O_2_ production. HEK-293 cells cotransfected with WT *DUOX1*, *DUOXA1*, DECTIN-1, and *PLCG2* constructs were stimulated with ionomycin or depleted zymosan, a β-glucan preparation from *Saccharomyces cerevisiae* that does not activate TLRs, resulting in measurable H_2_O_2_ production (Supplemental Figure 6). Substituting WT *DUOX1*, *DUOXA1*, or DECTIN-1 with patient variants or replacing *PLCG2* with GFP abrogated zymosan-induced H_2_O_2_ production (Figure 2D). *PLCG2* p.R268W supported H_2_O_2_ production in the overexpressed transfection system (data not shown), but primary PBMCs from patients with that variant did not upregulate TNF-α after β-glucan stimulation, suggesting a more complex underlying mechanism for this variant. Although cells transfected with DECTIN-1 Y238* did not produce DUOX1/DUOXA1–derived H_2_O_2_, individuals carrying DECTIN-1 Y238* had normal neutrophil superoxide production in both the homozygous and heterozygous states (Supplemental Figure 7).

Next, we wanted to assess whether these variants were specific risk factors for dissemination or infection. Within the validation cohort, there were 59 patients with chronic pulmonary coccidioidomycosis, defined as having treatment refractory (longer than 1 year) disease without extrapulmonary dissemination. Repeating the ancestry matching and 1:4 case to control ratio in DCM plus chronic disease, *CLEC7A* remained significantly overrepresented (*P* = 0.0041; OR, 2.058 [95% CI, 1.26–3.37]) with p.I223S (*P* = 0.0128; OR, 3.712 [95% CI, 1.32–10.426]) and p.Y238* (*P* = 0.0564; OR, 1.735 [95% CI, 0.985–3.058]) at or near significance. At the gene level, neither *PLCG2* nor *DUOX1/DUOXA1* were significant (*P* = 0.596 and 0.805, respectively), while at the variant level, PLCG2 p.R268W no longer reached significance (*P* = 0.0859).

We next expanded the analysis to include individuals with primary pulmonary disease, defined as those patients who sought treatment and cleared the infection with no further disease more than 1 year after treatment. We had 65 patients with primary pulmonary disease recruited from Arizona as well as 298 patients from the validation cohort. Including these individuals to look at infection risk rather than dissemination, we performed a 1:2 case to control ratio analysis. *PLCG2* was significantly overrepresented at the gene level in the discovery cohort (*P* = 0.0133) with p.R268W and p.N571S reaching variant-level significance (*P* = 0.025 and 0.0024, respectively). In contrast, no gene reached significance in the validation cohort and only PLCG2 p.N571S reached significance at the variant level (*P* = 0.036) as underrepresented. Taken together, these data suggest variants in the *CLEC7A*/*PLCG2*/*DUOX1/DUOXA1* pathway are not risk factors for primary infection but rather for control of infection (*CLEC7A*) and dissemination (*PLCG2/DUOX1/DUOXA1*).

**Figure 3:**
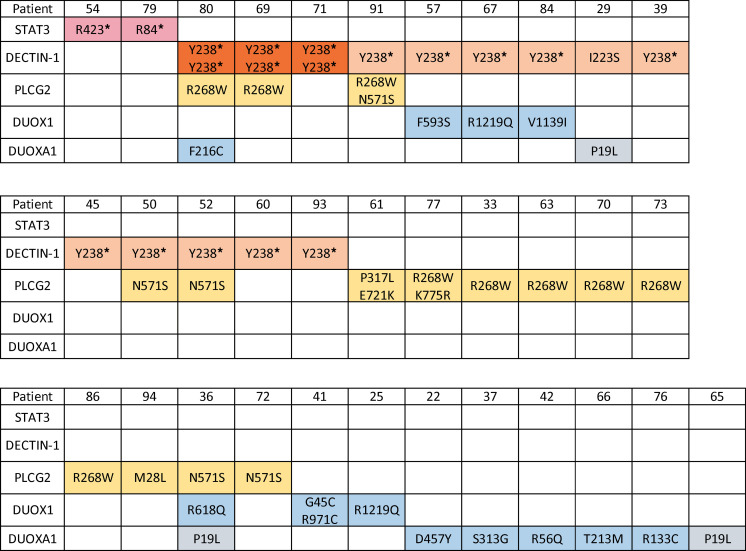
Exploratory cohort: 34 patients with DCM with identified fungal pattern recognition pathway or DUOX1 / DUOXA1 variants.

In total, STAT3 haploinsufficient mutations, variants in the DECTIN-1 fungal recognition pathway and H_2_O_2_-producing pathways involving DUOX1/DUOXA1 were found in 34 of 67 patients with DCM (50.7%) (Figure 3), spanning fungal recognition and response in both hematopoietic and nonhematopoietic compartments.

## Discussion

Population-level variants in fungal recognition and response genes affect both innate immune cells and pulmonary epithelia, the first responders to *Coccidioides* infection. These variants cause decreased TNF-α in response to fungal stimuli as well as decreased H_2_O_2_ production. To our knowledge, DECTIN-1–dependent production of H_2_O_2_ by DUOX1/DUOXA1 is previously unrecognized.

In the less than 1% of patients with *Coccidioides* infection who develop disseminated disease, exogenous immunosuppression is a major risk factor, as highlighted by the warnings for TNF-α biologics as a class. Consistent with this, *Tnfa*^–/–^ mice died more rapidly than WT mice after *Coccidioides* infection, with a median survival of 22.5 versus 70 days, and failed to form granulomata (34). Additionally, B6D2F1 mice, which are intrinsically resistant to *Coccidioides* infection, when treated with anti-TNF Abs for only the first 14 days after infection, had decreased survival and increased lung and spleen fungal burdens compared with isotype-treated controls (35). Last, mice with stable, controlled infection treated with anti-TNF Abs began dying as soon as 2 weeks after treatment, and treated mice exhibited significantly higher extrapulmonary fungal burdens (35). These data highlight the importance of TNF-α production and response both early in infection and for ongoing control of *Coccidioides* infection. Therefore, genetically impaired, PBMC TNF-α production after β-glucan stimulation in DCM is highly likely to be biologically relevant, based on human and mouse experience.

Development of mouse models for *Coccidioides* infection has been hampered by rapid lethality, limiting the ability to discriminate individual immune responses (34). Despite this, increased dissemination has been demonstrated for *Clec7a*^–/–^ mice (36) as well as for the TLR downstream signaling adaptor *Myd88*^–/–^ mice (36). Differences between susceptible C57BL/6 and resistant DBA mice have been attributed, in part, to a splice difference in *Clec7a* leading to a shortened DECTIN-1 surface receptor in C57BL/6 mice (37). Replacement of the short form with the complete long form confers resistance in C57BL/6 mice (37). These murine studies emphasize the importance of pattern recognition receptors in fungal response and control.

Several variants identified in our study have been previously implicated in fungal disease or immune dysregulation. Homozygous DECTIN-1 p.Y238* was reported in a family with mucocutaneous fungal infections (15), whereas heterozygosity in either donors or recipients was implicated in susceptibility to invasive aspergillosis after HSCT (16). Heterozygosity for DECTIN-1 p.I223S is associated with oropharyngeal candidiasis and reduced IFN-γ production after stimulation with heat-killed *Candida* *albicans* (38). A patient homozygous for p.I223S and DCM has previously been reported (39). Furthermore, a patient with compound heterozygous DECTIN-1 variants, p.I223S and p.Y238*, had severe, treatment-refractory infection with the far less virulent mold *Corynespora cassiicola* (40). *PLCG2* p.R268W was identified as a likely causal variant in a large, IBD GWAS (41), suggesting that this change may alter response to microbial antigens.

It is noteworthy that within our cohort, 26 patients with additional infections (Table 1) carried damaging variants in *CLEC7A, PLCG2*, or *DUOX1/DUOXA1*, compared with 6 of 27 patients without additional serious infections (Table 3) (*n* = 26 of 40 vs. 6 of 27; *P* = 0.001). Furthermore, 28 patients had viral infections, 19 of whom carried either *CLEC7A* or *PLCG2* variants, compared with 9 without variants (*n* = 19 of 34 vs. 9 of 33; *P* = 0.0258, Fisher’s exact test). Quintin et al. (42) demonstrated that β-glucan–induced training of monocytes from a patient deficient in DECTIN-1 failed to support increased cytokine release after subsequent challenge with various agonists. The recognition that β-glucan induced epigenetic changes can lead to trained immunity (43, 44) implies that the dampening of host response to β-glucan by these variants may affect patient immune response beyond acute fungal infections.

Although DUOX1/DUOXA1 variants have not previously been implicated in human disease, to our knowledge, airway-epithelial, DUOX1-derived H_2_O_2_ is sufficient to kill several oxidant-sensitive organisms including *Pseudomonas aeruginosa*, *Staphylococcus* *aureus*, *Burkholderia* *cepacia*, and *Hemophilus influenzae* (45). Hydrogen peroxide is also directly inhibitory to the growth of spherules (46). Beyond the antimicrobial effects of H_2_O_2_, alveolar macrophages respond to locally elevated H_2_O_2_ via the H_2_O_2_-conducting aquaglyceroporin AQP3 (47). Highlighting the signaling role of H_2_O_2_, pretreatment of alveolar macrophages with catalase suppressed chemokine production (47). Blocking transit of H_2_O_2_ using *Aqp3*^–/–^ mice reduced cytokine or chemokine production after immune activation of keratinocytes (48) or macrophages (47). Furthermore, *Aqp3*^–/–^ and *Duox1*^–/–^ mice had decreased leukocyte recruitment in models of allergic asthma (47, 49), and *Duox1*^–/–^ mice have increased susceptibility to influenza (30). More recently, Morris et al. (50) demonstrated a requirement for pulmonary-epithelial DUOX1 in acute house-dust-mite infection with significantly decreased IL-33 production 1 hour after infection. In a chronic infection model, macrophage-intrinsic Duox1 was required for macrophage recruitment and activation. These studies reinforce the importance of H_2_O_2_ generated by DUOX1/DUOXA1 at both the pulmonary epithelial surface and in alveolar macrophages. We have demonstrated that fungal recognition through DECTIN-1 leads to production of H_2_O_2_. In the lung, this may be pulmonary epithelia derived but available to nearby alveolar macrophages via AQP3, macrophage intrinsic, or both, leading to enhanced signaling and release of cytokines and chemokines, all of which have been demonstrated in other systems (47, 48, 50–52). Despite the ability of DECTIN-1 to activate NADPH oxidase activity in both DUOX1/DUOXA1 and NOX2/gp91*^phox^* systems, patients with chronic granulomatous disease due to defects in the NOX2 complex do not have increased risk of infection from *Coccidioides* (53), and gp91*^phox^* mice have normal resistance to intranasal *Coccidioides* infection (54, 55).

Genetic variants identified by GWAS have been implicated in numerous studies of broad health risks such as heart disease, obesity, and dementia. Specific variants present in the population that confer increased risk of tuberculosis [TYK2 P1104A (56)], West Nile virus [CCR5Δ32 (57)], schizophrenia [loss-of-function SETD1A mutations (58)], or protection from HIV [CCR5Δ32 (59)] have been previously reported. Similar to these studies, we demonstrate the roles of common population variants that are benign unless the carrier is infected with *Coccidioides*, a geographically isolated, pathogenic organism.

In summary, early defects in innate fungal recognition and response are critical to the host control of the pathogen *Coccidioides;* defects in these pathways contribute to the development of severe coccidioidomycosis, a predisposition only apparent in the corresponding locale. These mostly population-based variants define a critical set of previously unrecognized gene and environment interactions.

## Methods

### Patients and control participants.

Patients with biopsy-proven DCM or with limited pulmonary infections but without comorbidities predisposing to DCM provided informed consent to be enrolled in studies approved by the IRBs of the National Institute of Allergy and Infectious Diseases or University of Arizona, respectively. Samples were obtained from healthy donors from the NIH blood bank. DNA was isolated from whole blood or saliva. PBMCs were isolated using density centrifugation from heparinized whole blood.

Our validation cohort consisted of patients known to have coccidioidomycosis who were treated at UCD, each of whom provided informed consent to be enrolled in studies approved by the UCD IRB. Patient charts were reviewed and adjudicated on the basis of the type of coccidioidal infection. Those classified as having had primary pulmonary infection had no evidence of disease recurrence 2 years after stopping antifungal therapy. Those with chronic pulmonary infection exhibited ongoing clinical and radiographic evidence of coccidioidomycosis after more than 1 year of antifungal therapy. Disseminated infection was defined as disease outside the thorax. In all cohorts, exclusion criteria included pregnancy, abnormal complete blood cell count, positive for HIV, evidence of rheumatologic or other potentially immunocompromising disease, immunosuppressant medication use, or evidence of invasive fungal disease other than coccidioidomycosis.

### Whole-exome sequencing and analysis.

Whole-exome sequencing in the discovery DCM and Arizona pulmonary coccidioidomycosis cohorts was performed using the Ion Torrent AmpliSeq RDY Exome Kit (Life Technologies) and the Ion Chef and Proton instruments (Life Technologies). Briefly, 100 ng of genomic DNA was used as the starting material for the AmpliSeq RDY Exome amplification step, following the manufacturer’s protocol. Library templates were clonally amplified and enriched using the Ion Chef and the Ion PI Hi-Q Chef Kit (Chef package version IC.4.4.2, Life Technologies), following the manufacturer’s protocol. Enriched, templated Ion Sphere Particles were sequenced on the Ion Proton sequencer using the Ion PI chip v3 (Life Technologies). Read mapping and variant calling were performed using Ion Torrent Suite software, version 4.4.2. Sequencing reads were mapped against the University of California, Santa Cruz, hg19 reference genome using the Torrent Mapping Alignment Program map4 algorithm. SNPs and insertions and deletions were called by the Torrent Variant Caller plugin (version 4.414-1) using the Generic-Proton-Germ Line: Low Stringency configuration. Only reads that were unambiguously mapped were used for variant calling. Variants were annotated using ANNOVAR (http://annovar.openbioinformatics.org/).

The validation cohort was sequenced using the Nextera Rapid Capture Exome Kit. Samples were sequenced at the Broad Institute on Illumina HiSeq sequencers using the Illumina Nextera exome capture kit. Each sample’s sequencing reads were aggregated into a BAM file and processed through a pipeline based on the Picard set of software tools. The BWA aligner mapped reads onto the human genome build 37 (hg19). SNP and insertions and deletions were jointly called across all samples using Genome Analysis Toolkit (60) *HaplotypeCaller* package, version 3.4, to produce a version 4.1 variant callset file. Variant call accuracy was estimated using the Genome Analysis Toolkit Variant Quality Score Recalibration approach.

### Genomic boundaries for variants.

Variants falling within the following hg19 regions were extracted from the variant callset files of the validation cohort and 1000G: *CLEC7A* chr12:10269376-10282868; *PLCG2* chr16:81812899-81991899; *DUOX1/DUOXA1* chr15:45409564-45457774; *STAT3* chr17:40,465,343-40,540,513. Because DUOX1 and DUOXA1 are adjacent on the chromosome, a single interval was used to capture both genes. Variants were annotated using CADD version 1.6 (61) (https://cadd.gs.washington.edu/score).

### Variant nomenclature.

The following transcripts were used for variant nomenclature: *STAT3* NM_003150, *CLEC7A* NM_197947, *PLCG2* NM_002661, *DUOXA1* NM_144565, and *DUOX1* NM_017434.

### Cytokine production.

PBMCs were plated in 96-well plates at 5 × 10^5^ cells/well in 100 mL of complete RPMI medium and stimulated for 24 hours with 100 mg/mL purified, particulate β-glucan (62) or 100 ng/mL LPS (Invitrogen). Supernatants were harvested and cytokine levels measured by ELISA (R&D Systems). Normalized cytokine levels were compared using an unpaired *t* test (GraphPad Prism).

### Transfection studies and H_2_O_2_ measurements.

WT, HA-tagged DUOX1 and V5-tagged DUOXA1 expression constructs (29) were used for site-directed mutagenesis to create patient-identified variants. Flp-In-293 cells stably expressing WT DUOXA1 or DUOX1 were transfected (Fugene HD, Promega) with HA-tagged DUOX1 or V5-tagged DUOXA1 expression constructs, respectively. Alternatively, HEK-293 cells were cotransfected with DUOX1, DUOXA1, DECTIN-1, PLCG2, or empty vectors. At 48 hours after transfection, cells were stimulated with 1 μM ionomycin (Invitrogen) or 100 μg of depleted Zymosan (Invivogen). H_2_O_2_ release was continuously measured (Luminoskan Ascent plate reader, Thermo Fisher Scientific). using luminol and HRP (Sigma-Aldrich) for 60 minutes. Unstimulated, transfected cells were harvested for immunoblot analysis.

### Immunoblotting.

Transfected cells were lysed in RIPA buffer with protease inhibitors. A total of 20 μg of protein per sample was denatured and loaded on a 10% polyacrylamide gel. After electrophoresis, proteins were transferred to nitrocellulose membranes, blocked for 1 hour in TBS plus Tween with 5% powdered milk, then probed with specific Abs. Abs used were anti-HA (Covance; 1:1000), anti-V5 (Thermo Fisher Scientific, catalog R960-25; 1:1000), and anti–HSP-90 (Santa Cruz, catalog sc-13119; 1:3000). Blots were imaged using IBRIGHT FL100 imaging system (Thermo Fisher Scientific).

### Confocal microscopy.

Paraffin sections (5 μm) were incubated at 60°C for 60 minutes, dewaxed in xylene, and rehydrated in ethanol. Antigen retrieval was performed at pH 7.4 in 0.00356 M citraconic acid solution in a steamer for 40 minutes. Slides were blocked in a solution of 1 M Tris, 0.1% Tween 20, 0.5% 40% to 50% gelatin from cold-water fish skin (Sigma-Aldrich), and 0.1% of 0.00356 M citraconic acid solution. Slides were stained with the following Abs: Dectin-1 (1:20; clone RH1, BioLegend, catalog 144302, lot B218624); LAMP-1 (1:50; Abcam, ab208943, lot GR3213103-10) followed by fluorescent conjugated secondary Abs (anti-rat AF647, Invitrogen, catalog A11077; anti-rabbit AF488, Jackson ImmunoResearch, catalog 711-545-152; both 1:100) for 2 hours at room temperature. Chitin and cellulose (Calcofluor white reagent B, Remel, R40015, lot 179886), TO-PRO-3 iodide (Invitrogen, lot 2069619) was used for nuclear stain and Calcofluor white reagent B (Remel) was used to stain *Coccidiodes* and mount slides. Images were acquired on a Leica SP5 inverted confocal microscope equipped with HyD hybrid detectors.

### Image analysis.

Images were deconvolved using Huygens Professional (Scientific Volume Imaging), and colocalization was analyzed using the coloc feature of Imaris 9.7.2 (Oxford Instruments).

### Case–control matching.

PCA of the genotype data was performed using ~10,000 ancestry-informative variants, after which 4 population controls from the 1000G (*n* = 2504) were selected for each DCM case using the R package *Optmatch* (https://cran.r-project.org/web/packages/optmatch/index.html), based on PC1–PC5.

### Statistical analysis of variant burden.

A logistic regression model for the binary response of disseminated disease status was used to estimate the OR of variant burden in our genes of interest, adjusting for principal components of ancestry background. Three gene association tests (*CLEC7A,* *PLCG2,* and *DUOX1/DUOXA1*) were performed. Predicted damaging variants were selected on the basis of gnomAD_controls_AF_popmax <0.1, CADD (version 1.6) phred score ≥20, and with PASS filter status in the variant callset file. The association was first examined in the discovery-matched cohort and the significant gene was tested in the validation cohort. A *P* value < 0.05 (2 tailed) was considered significant and a gene OR of greater (less) than 1 is interpreted as indicating positive (negative) association. Bonferroni adjustment was used to correct for multiple testing. Association tests were then performed for recurring variants.

### Data availability.

Whole-exome sequence data are available in dbGaP, study accession phs002881.v1.p1 (Discovery DCM) and phs002995.v1.p1 (Arizona pulmonary). Validation cohort data are available as supplementary materials, including disease presentation and ancestry (Supplemental Table 1), PCA of the genotype data from Optmatch (Supplemental Table 2), and variants identified in CLEC7A, PLCG2, and DUOX1/DUOXA1 (Supplemental Table 3).

### Statistics.

Statistical analyses were performed using GraphPad Prism 8.0 software. Statistical tests used include Fisher’s exact test, Brown-Forsythe’s and Welch’s ANOVA with Dunnett’s T3 multiple comparisons test, ordinary one-way ANOVA, and Dunnett’s multiple comparisons test. Data were analyzed by unpaired *t* tests. In cases where multiple data sets were analyzed, 1- or 2-way ANOVA was used with Dunnett’s correction. In all cases, *P* < 0.05 was considered significant.

### Study approval.

All patients provided informed consent to participate in protocols approved by the IRBs of the National Institute of Allergy and Infectious Diseases (discovery cohort, healthy volunteers), University of Arizona (pulmonary cocci), or UCD (validation cohort).

## Author contributions

SMH and JNG designed the project. APH, GVR, RCB, LFS, TLL, MSL, and SMH designed the research studies. APH, AK, CCA, GC, LS, JVD, SDR, JEN, JS, and LFS conducted experiments. APH, JG, EK, AJO, LS, JBL, DAP, NP, YAL, J. Frelinger, TLL, and MSL analyzed data. DY provided reagents. JD, AC, TP, DJB, ERS, SEW, FMD, DALP, AFF, CSZ, DBK, KNO, J. Fierer, GRT, JNG, MSL, and SMH were responsible for patient care and accrual. Resources and supervision were provided by RCB, HDH, TLL, GRT, JNG, MSL, and SMH. APH and SMH wrote the manuscript, all authors participated in interpreting the data and editing the manuscript.

## Supplementary Material

Supplemental data

Supplemental tables 1-3

## Figures and Tables

**Table 2:**
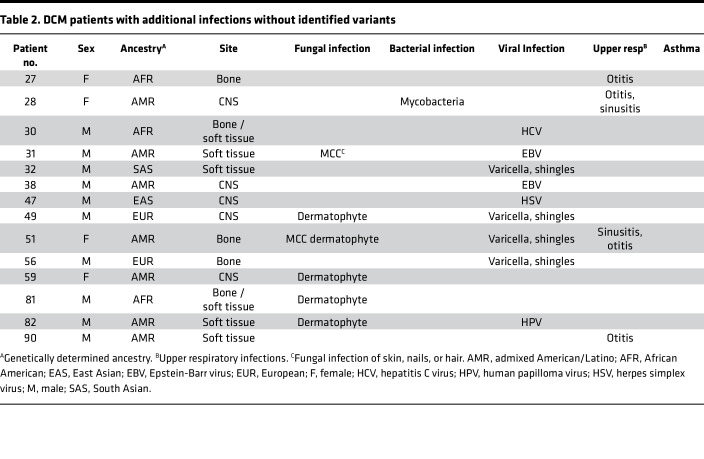
DCM patients with additional infections without identified variants

**Table 1:**
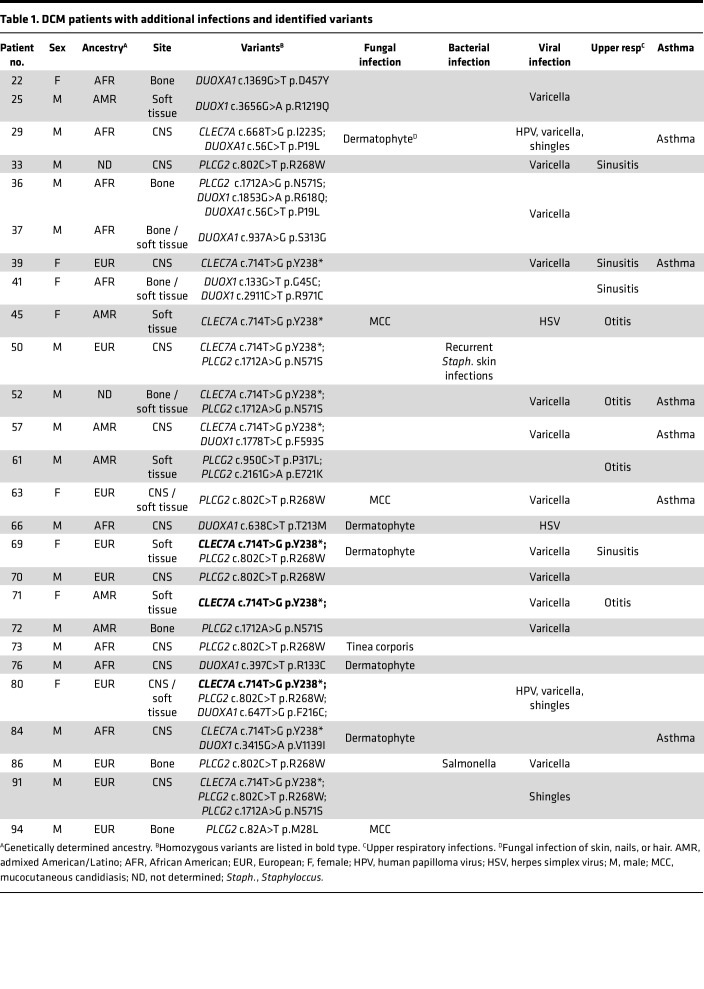
DCM patients with additional infections and identified variants

**Table 3:**
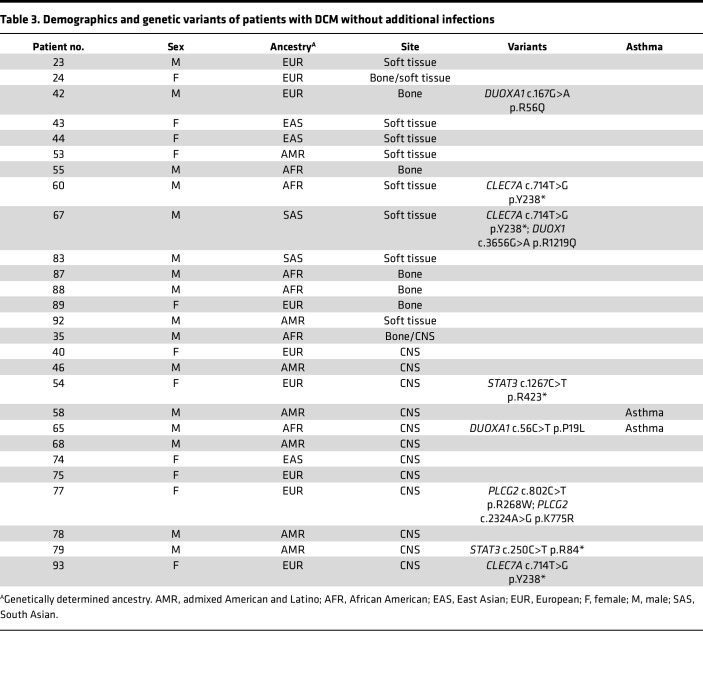
Demographics and genetic variants of patients with DCM without additional infections

## Version 1 09/27/2022

In-Press Preview

## Version 2 11/22/2022

Electronic publication
