# Supplementary material for: Immunogenetics associated with severe coccidioidomycosis
Source: JCI Insight. 2022 Nov 22;7(22):e159491. doi: 10.1172/jci.insight.159491 (PMC9746810; doi:10.1172/jci.insight.159491)
Supplement: Supplemental data [file jciinsight-7-159491-s273.pdf]

Supplementary Material:

Title: Immunogenetics associated with severe coccidioidomycosis

Table of Contents

Supplemental Figure S1. Fold enrichment of CLEC7A, c.714T>G; p.Y238* genotype in DCM compared to gnomAD……………………………………………………………………………..……………….2

Supplemental Figure S2. Structure of murine DECTIN-1 C-type lectin domain.................3

Supplemental Figure S3. PBMC cytokine production from healthy controls or DCM patients with and without DECTIN-1 pathway variants……..……………………………………..….4

Supplemental Figure S4. Coccidioidomycosis disease presentation by ancestry…………..…6

Supplemental Figure S5. Log_{10} DUOX1/DUOXA1 variant frequency in DCM cohorts and gnomAD...7

Supplemental Figure S6. DUOX1/DUOXA1 produces H_2O_2 after DECTIN-1 engagement……..........8

Supplemental Figure S7. Normal neutrophil ROS in DECTIN-1 Y238* individual…………..…..9

Supplemental Table S4. Overrepresentation of DUOX1/DUOXA1 variants by ancestry....10

Supplemental Figure S1. Fold enrichment of *CLEC7A*, c.714T>G; p.Y238* genotype in DCM compared to gnomAD.

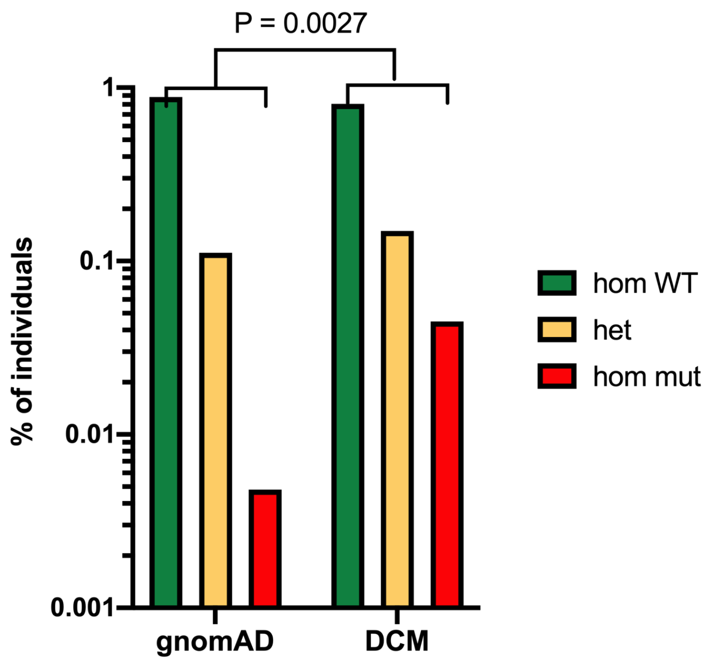

Frequency of CLEC7A, c.714T>G genotype in DCM normalized to gnomAD database frequency. Using frequency of each genotype in gnomAD as 1, there is a significant increase in the proportion of the cohort homozygous for Y238* (P=0.0027 Fisher's Exact comparing homozygous genotypes between DCM and gnomAD).

Supplemental Figure S2. Structure of murine DECTIN-1 C-type lectin domain

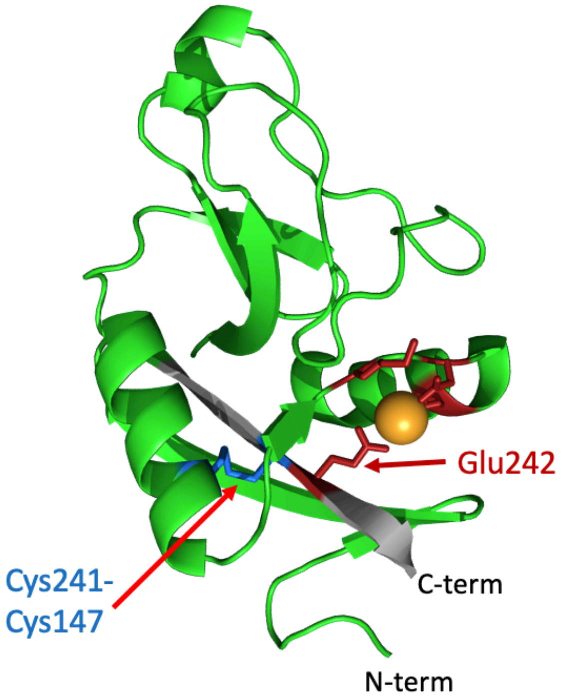

Murine DECTIN-1 C-type lectin domain (PDB ID 2BPE)[19]. Amino acids deleted by Y238* are shown in grey, residues forming disulfide bonds (blue) and binding Ca2+ (red) are highlighted.

Supplemental Figure S3. PBMC cytokine production from healthy controls or DCM patients with and without DECTIN-1 pathway variants.

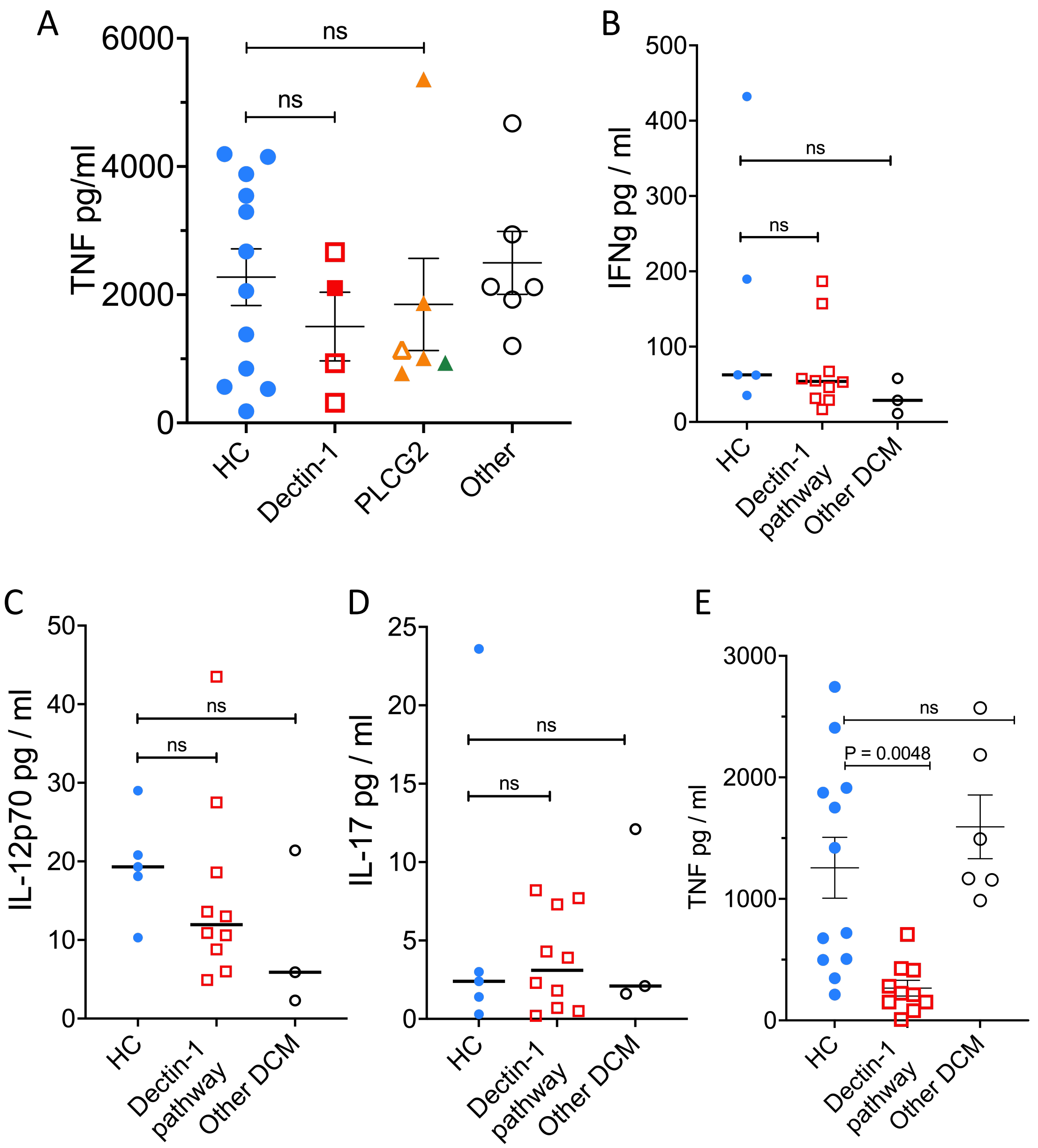

A. LPS-induced TNF production was measured by ELISA. Individuals are grouped as healthy controls, DECTIN-1 or PLCG2 variants or "Other" for those DCM patients without an identified variant. B. β-glucan induced IFNγ production. Individuals are grouped as in A. C. β-glucan induced IL-12p70 production. Individuals are grouped as in A. D. β-glucan induced IL-17

production. Individuals are grouped as in A. D. Data from Figure 1F grouping DCM patients with DECTIN-1 pathway variants together. P values calculated using Brown-Forsythe and Welch ANOVA.

Supplemental Figure S4. Coccidioidomycosis disease presentation by ancestry

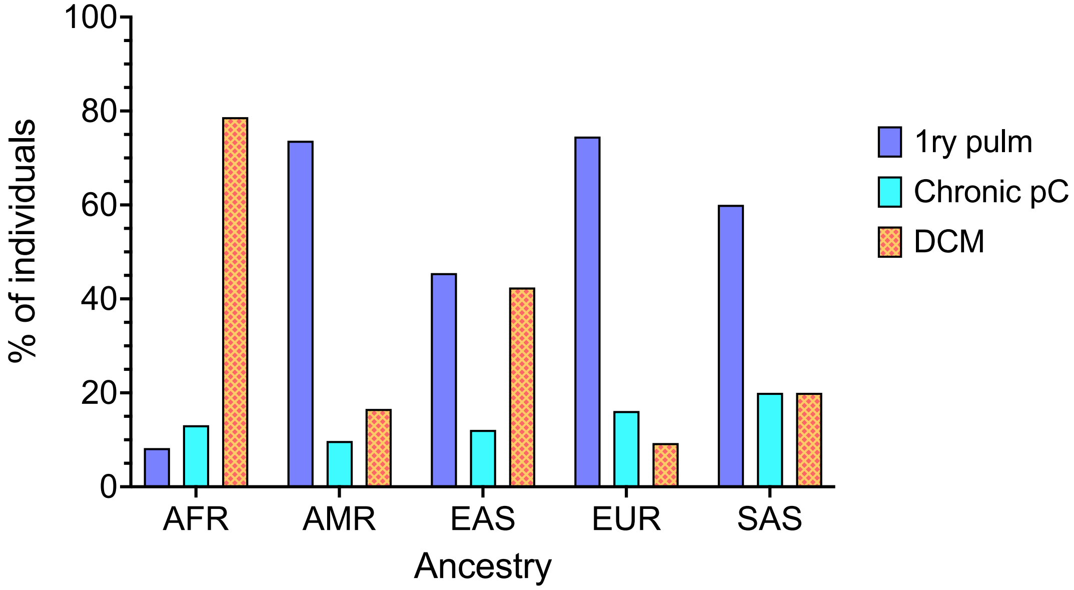

Disease presentation of individuals from validation cohort grouped by genetically determined ancestry. Presentations include primary pulmonary (1ry pulm), chronic pulmonary (Chronic pC) and disseminated disease (DCM).

Supplemental Figure S5. Log_{10} DUOX1/DUOXA1 variant frequency in DCM cohorts and gnomAD

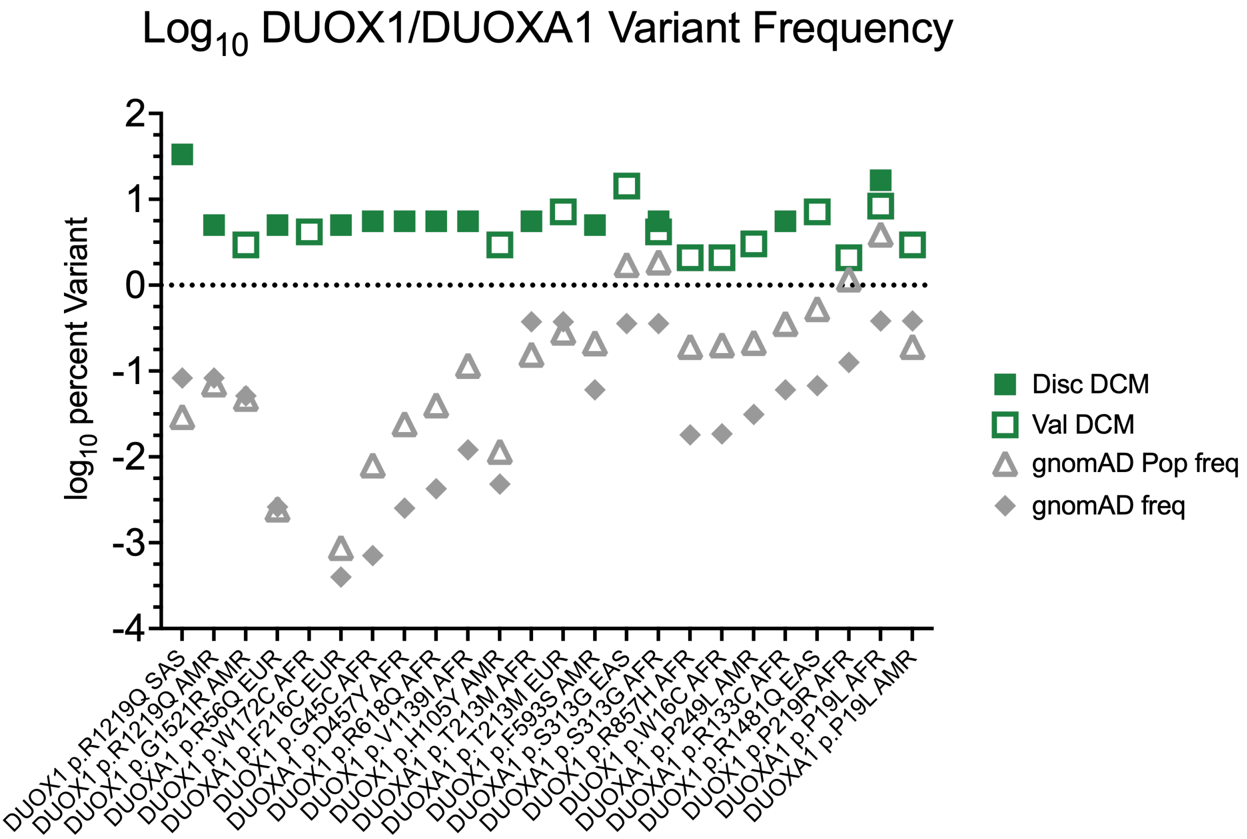

Variant frequencies from discovery (Disc DCM) and validation (Val DCM) compared to gnomAD v2.1 ancestry-specific population frequency (gnomAD Pop freq) or total gnomAD v2.1 samples (gnomAD freq).

Supplemental Figure S6. DUOX1/DUOXA1 produces H_2O_2 after DECTIN-1 engagement.

A.

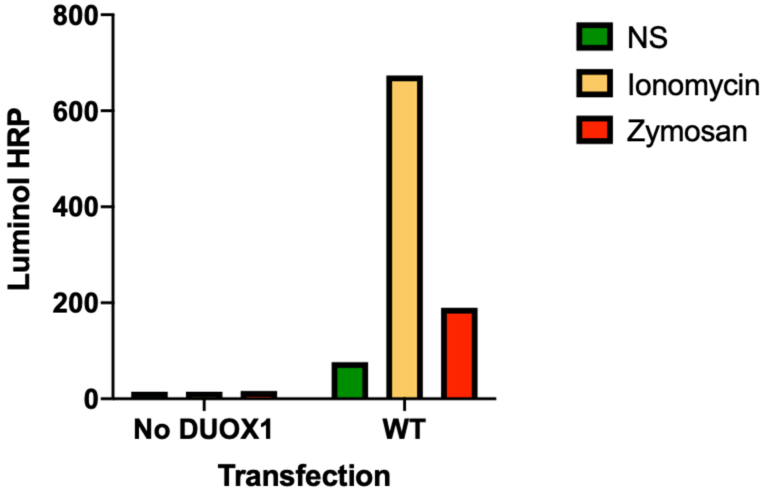

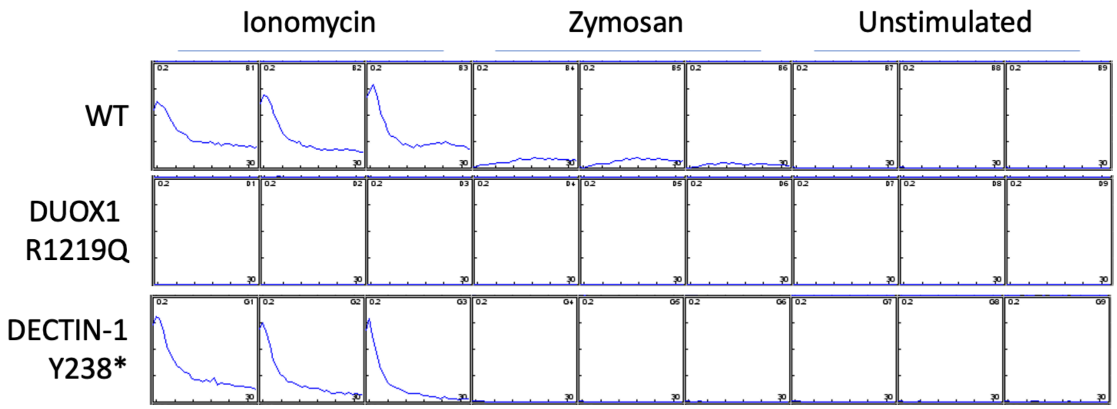

B.

HEK-293 cells were transiently transfected with DUOX1, DUOXA1, DECTIN-1 and PLCG2 expression constructs. 48 hours later cells were stimulated or not with Ionomycin (100 ng) or depleted Zymosan (100 μg). H_2O_2 production was measured for 60 minutes. A. Cumulative H_2O_2 production from transfected cells. Data is average of triplicate wells from one representative experiment. B. Kinetics of H_2O_2 production. Graphs show kinetics of H_2O_2 production in triplicate wells from one representative experiment.

Supplemental Figure S7. Normal neutrophil ROS in DECTIN-1 Y238* individual

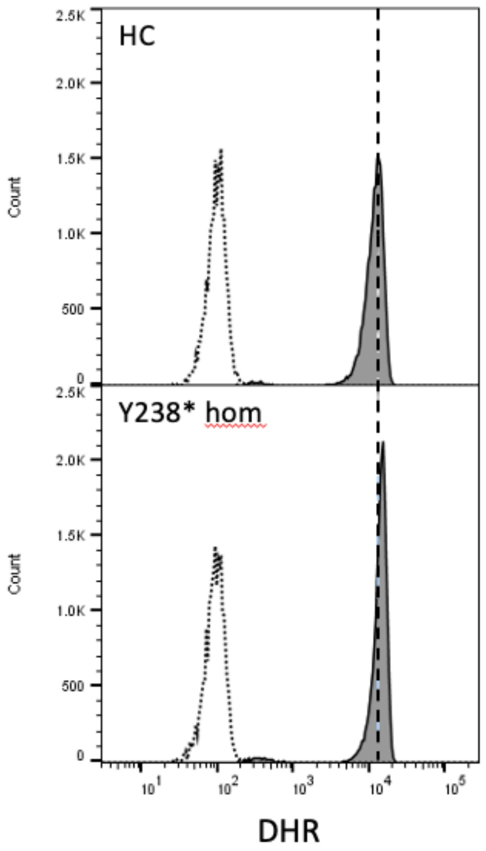

Neutrophil dihydrorhodamine oxidation (DHR) from a healthy control (HC) or an individual homozygous for DECTIN-1 Y238*. Neutrophil populations were gated using forward and right-angle light scattering. Open histograms – neutrophil ROS production with buffer under basal conditions; solid histograms – neutrophil ROS production in response to PMA (400 ng/mL). The vertical dashed line represents the peak of PMA-treated neutrophils. Representative histogram from one of five DECTIN-1 Y238* patients evaluated.

Supplemental Table S4. DUOX1/DUOXA1 variants overrepresentation by ancestry

Gene	Variant	CADD	Ancestry	Disc DCM	Val DCM	gnomAD Pop count*	P value@	gnomAD Pop freq&	gnomAD freq$	LOF
DUOX1	p.R1219Q	23	SAS	1/3		9/15308	**0.002**	0.000294	0.0008273	y#
DUOX1	p.R1219Q	23	AMR	1/20		25/17720	**0.0289**	0.0007054	0.0008273	y
DUOX1	p.G1521R	28.6	AMR		1/34	17/17717	**0.0339**	0.0004798	0.000514	y
DUOXA1	p.R56Q	33	EUR	1/20		3/61134	**0.0013**	0.00002454	0.00002611	y
DUOX1	p.W172C	27.8	AFR		2/48	0/20737	**<0.0001**	0	0	
DUOXA1	p.F216C	23.7	EUR	1/20		1/56870	**0.0007**	0.000008792	0.000003977	
DUOX1	p.G45C	27.3	AFR	1/18		2/12474	**0.0043**	0.00008017	0.00000708	
DUOXA1	p.D457Y	21.9	AFR	1/18		6/12378	**0.0101**	0.0002424	0.00002525	
DUOX1	p.R618Q	19.8	AFR	1/18		10/12476	**0.0157**	0.0004008	0.00004247	
DUOX1	p.V1139I	17.44	AFR	1/18		29/12473	**0.0424**	0.001163	0.0001204	
DUOX1	p.H105Y	24.9	AMR		1/34	4/17268	**0.0098**	0.0001158	0.0000482	
DUOXA1	p.T213M	27.3	AFR	1/18		39/12472	*0.0561*	0.001564	0.003756	
DUOXA1	p.T213M	27.3	EUR		1/15	362/64553	*0.0811*	0.002835	0.003756	
DUOX1	p.F593S	22.4	AMR	1/20		75/17712	*0.0824*	0.002117	0.0006047	
DUOXA1	p.S313G	23.1	EAS		2/14	344/9976	*0.0827*	0.01734	0.003572	
DUOXA1	p.S313G	23.1	AFR	1/18	2/48	457/12473	0.4898	0.01859	0.003572	
DUOX1	p.R857H	24.2	AFR		1/48	48/12484	0.1717	0.001922	0.0001803	
DUOX1	p.W16C	22.2	AFR		1/48	50/12505	0.1778	0.002007	0.0001851	
DUOXA1	p.P249L	21.8	AMR		1/33	76/17713	0.1375	0.002145	0.0003113	
DUOXA1	p.R133C	25.2	AFR	1/18		89/12471	0.1221	0.003568	0.0006048	
DUOX1	p.R1481Q	27.6	EAS		1/14	106/9977	0.14	0.005312	0.0006753	

DUOX1	p.P219R	24.6	AFR		1/48	189/8063	>0.9999	0.01178	0.001257
DUOXA1	p.P19L	22.1	AFR	3/18	4/48	954/12453	0.1557	0.03919	0.003831
DUOXA1	p.P19L	22.1	AMR		1/34	68/17641	0.1246	0.001927	0.003831

* Number of variant positive individuals / Number of individuals sequenced for given ancestry-specific population
@ Fishers exact test for population frequency between DCM cohort and ancestry-specific population in gnomAD v2.1. $P < 0.05$ denoted in **bold**, $P < 0.10$ in *italics*.
[&] Frequency of variant alleles in ancestry-specific population in gnomAD v2.1
[$] Frequency of variant alleles in gnomAD v2.1
Demonstrated loss of function in heterologous overexpression assay
